# Paralysis of the Intestines after Abdominal Operations

**Published:** 1898-05

**Authors:** 


### Abstracts and Selections.

### Paralysis of the Intestines after Abdominal Operations.

Engstrom (*Contrabl. fur Gynak.*, Sept. 11, 1897,) says that paralysis of the intestines which follows laparotomy, sometimes producing death, does not always result from sepsis. He cites four cases in which death followed operation after fifty-seven hours, seven, eight and ten days respectively. In these four cases there was not the least trace of peritonitis, which there would certainly have been if death had been due to sepsis after such an interval of time. Moreover, in one case the contents of the amdominal cavity were examined bacteriologically one hour after death and were found to be absolutely sterile. According to the author, a careful stimulation and nutrition of the patient, if necessary, per rectum, is a most important factor in preserving life.—*Medical News.*

